# Alteration of Metabosensitive Afferent Response With Aging: Exercised versus Non-exercised Rats

**DOI:** 10.3389/fnagi.2018.00367

**Published:** 2018-11-12

**Authors:** Guillaume Caron, Patrick Decherchi, Tanguy Marqueste

**Affiliations:** Aix-Marseille Univ, CNRS, ISM, Equipe Plasticité des Systèmes Nerveux et Musculaire, Faculté des Sciences du Sport, Marseille, France

**Keywords:** age, fatigue, flexor, extensor, electrophysiology, muscle

## Abstract

This study was designed to evaluate the effect of aging on the activity of metabosensitive afferent fibers (thin muscle afferents from group III and IV) and to determine if physical activity performed at old age may influence the afferent discharge. Afferents from *tibialis anterior* and *soleus* muscles were recorded on non-exercised rats aged of 3, 6, 12, and 20 months and on animals aged of 12 and 20 months performing a daily incremental treadmill exercise protocol during the last 8 weeks preceding the recordings. Metabosensitive afferent fibers were activated with potassium chloride (KCl) and lactic acid (LA) injections into the blood stream or by muscle electrically-induced fatigue (EIF). Results indicated that aging is associated to a decrease in the magnitude of the responses to chemical injections and EIF. Unfortunately, physical activity did not allow restoring the metabosensitive afferents responses. These results indicate an alteration of the thin afferent fibers with aging and should be taken into account regarding the management of muscle fatigue and potential alterations of exercise pressor reflex (EPR) occurring with aging.

## Introduction

Muscle contractions enhance neuronal adjustments regulated by activation of afferents originating from activated muscles. Indeed, among muscle afferents, metabosensitive fibers from groups III and IV are activated by metabolic, mechanical and thermal modification of their receptive fields occurring during and after repetitive contractions (Laurin et al., 2015). Metabolic agents such as lactic acid (LA) and potassium chloride (KCl), and electrically induced exercise (EIF) are also known to be specific activators of these thin metabosensitive afferents fibers (Rotto and Kaufman, 1988; Victor et al., 1988; Decherchi et al., 1998, 2001). When stimulated, these afferents change the motoneurons excitability in the spinal cord (Dousset et al., 2004; Laurin et al., 2010). Furthermore, these afferents are responsible for the sensation of muscle pain (Mense, 2009) and also project to brainstem level to induce an exercise pressor reflex (EPR), a neural drive originating from skeletal muscles that result in an increase in the sympathetic activity associated with an up-regulation of both heart and ventilation rate, and arterial blood pressure (McCloskey and Mitchell, 1972).

Neuromuscular system is highly malleable at young age, but this plasticity tends to be reduced at old age. Indeed, muscle strength progressively declines (Vandervoort and McComas, 1986) mostly because of sarcopenia, a phenomena resulting to a decrease in the number of type I and II muscle fibers, and to a type II muscle fiber atrophy (Lexell, 1995). Because muscle mass and phenotype were shown to take part in determining the magnitude of the EPR and the response of the metabosensitive afferents (Iwamoto and Botterman, 1985; Wilson et al., 1995; Xing et al., 2008; Caron et al., 2015), sarcopenia could be at the origin of a down regulation of the EPR and a decrease in the metabosensitive activity. This was suggested by some authors reporting a down regulation of the EPR with aging (Markel et al., 2003; Houssiere et al., 2006) but disputed by other showing that the EPR was maintained with aging (Ng et al., 1994; Greaney et al., 2013). Nevertheless, a recent study underlined an age-related alteration of the contribution of the metabosensitive muscle afferents to the hemodynamic response to exercise (Sidhu et al., 2015). Finally, because it was described that a period of inactivity induces a lower EPR during leg isometric exercise and during post-exercise ischemia (Kamiya et al., 2004) and an abnormal EPR in many forms of hypertension, heart failure or muscular dystrophy, exercise training has been proposed to restore the EPR (Murphy et al., 2011; Smith et al., 2014).

Thus, the aim of the present study was to record, with electrophysiological tools, the response of metabosensitive afferent fibers originating from *tibialis anterior* and *soleus* muscles to chemical injections of LA and KCl, EIF at 3, 6, 12 and 20 months of age in non-exercised rats, and at 12 and 20 months of age after 8 weeks of incremental treadmill running protocol. We hypothesized that metabosensitive afferents response is altered with aging and that repeated physical activity could reverse this alteration.

## Materials and Methods

### Animals and Ethical Approval

Sixty-nine male Sprague Dawley rats (Janvier Lab^®^, France) were housed in smooth-bottomed plastic cages at 22°C in a room maintain on a 12-h light/dark cycle. Food (Safe^®^, France) and water were available *ad libitum*. Forty-eight were randomly allocated into 4 groups according to their age: 3 months (3 M, *n* = 12), 6 months (6 M, *n* = 12), 12 months (12 M, *n* = 12) and 20 months (20 M, *n* = 12), and allowed to age until electrophysiological recordings. Two other groups performed treadmill training during 8 weeks before the electrophysiological session: 12 months old (12M-EXE, *n* = 10) and 20 months old (20M-EXE, *n* = 11). The exercise effects in the 12M-EXE and 20M-EXE groups were compared to the 12 M and 20 M animals from the non-exercised groups, respectively.

All procedures outlined in this study were approved (license n°A 13.013.06) by the animal ethics committee of *Aix-Marseille University* (AMU) and *Centre National de la Recherche Scientifique* (CNRS). All experiments were performed in accordance with the recommendations listed in the Guide for Care and Use of Laboratory Animals (U.S. Department of Health and Human Services, National Institutes of Health) and the European Community’s council directive of 24 November 1986 (86/609/ EEC).

### Exercise Training Protocol

Rats were first familiarized with the treadmill for 1 week. Then, animals were trained 3 times per week on a treadmill with a progressive 8 weeks protocol inspired from Pasini et al. and previously described (Pasini et al., 2012; Caron et al., 2016). Briefly, duration of the exercise in the first and the second week was 10 and 20 min, respectively, with a running speed fixed at 13.5 m/min. For the next 3 weeks, running time and speed were progressively increased to reach at the 5th week a duration of 50 min at 15 m/min speed. Finally, for the last 3 weeks, exercise was performed for 60 min and speed was increased up to 18 m/min until the 8th week. All animals ran steadily on the treadmill. Rats were anesthetized for electrophysiological recordings within the 48h after the end of the exercise-training protocol.

### Electrophysiological Recordings

When animals reach the age of their respective group, they were anesthetized with urethane (1.1 g.kg^-1^ i.p.), and atropine (1 mg.kg^-1^, i.p.) was administered to reduce airway secretions. The surgery and the afferent recordings were performed as previously described (Decherchi et al., 1998; Caron et al., 2014, 2015). Briefly, a catheter was inserted into the right femoral artery to let the blood flow freely to the left lower limb muscles and pushed up to the fork of the abdominal aorta in order to transport chemicals (i.e., potassium chloride [KCl] and lactic acid [LA]) to the controlateral muscle. Branches of nerves innervating the *soleus* and *tibialis anterior* muscles were dissected free from surrounding tissues, cut distally, immersed in paraffin oil and placed on two pairs of tungsten bipolar cuff electrodes for afferents recordings. The knee and ankle were firmly held by clamps on a horizontal support in order to avoid disturbing movements and to maintain the 90° knee joint angle during electrical muscle stimulation inducing an EIF. Animal temperature was maintained between 36 and 37°C with a blanket controlled by a rectal temperature probe.

Activities originating from the nerves were recorded and referred to a ground electrode implanted in a nearby muscle, amplified (1–100 K), filtered (30 Hz to 10 kHz) with a differential amplifier (P2MP^®^ SARL, France) and fed into an amplitude window discriminators (P2MP^®^ SARL) analyzing action potentials. The discriminators separated action potentials on the basis of their amplitude and provided an output pulse for the desired signal. For every waveform peak that appears within the window aperture (crossing the lower level of the windows) set by the user, a rectangular pulse was generated. Signals exceeding the upper level of the window (crossing the lower and upper levels) were not considered. Multiplexing the input signal and window discriminators provided convenient oscilloscope visualization and ease of setting up the experiment. It eliminated adjusting the oscilloscope levels for drift. In absence of any movement, only metabosensitive afferent fibers exhibiting spontaneous tonic low frequency baseline activity were active (Decherchi et al., 1998). Thus, metabosensitives afferent activities were selected according to their action potential amplitudes which were higher than the background noise. The output provided noise-free tracings (discriminated units) on which action potentials were displayed on a computer and then counted using data analysis system (Biopac MP150^®^ and AcqKnowledge^®^ software, United States) at 1s intervals (in Hz). Baseline discharge was calculated during the 1-min period preceding injections or EIF, and its change was measured following specific activations. Afferent response variations were expressed in percentage of the corresponding baseline discharge rate (F_impulses_.s^-1^, % of baseline activity).

In a first step, distinct concentrations of KCl (1, 5, 10, and 20 mM / 0.5 ml) and LA (0.5, 1, 2, and 3 mM / 0.1 ml) were randomly injected into the catheter and were washed with 0.1 ml of normal saline. Each injection was separate by 10 min of recovery in order to let the afferent activity go back to its baseline activity.

In a second step, after a 30-min resting period, a 3-min EIF was performed. For this purpose, rhythmic muscle contractions were produced by a stimulator (Grass S88K^®^, United States) delivering rectangular pulse trains to a pair of steel electrodes placed on the muscle surface (pulse duration: 0.1 ms; frequency: 10 Hz, i.e., 5 shocks in each 500 ms train; duty cycle: 500/1000 ms). The voltage used was 20% higher than that used to elicit a maximal contraction.

At the end of the experiments, animals were sacrificed by an overdose (3 ml, i.a) of sodium pentobarbital solution (60 mg.kg^-1^, Nembutal^®^, Sanofi Santé Animale, France).

### Statistics

Data processing was performed using Sigmaplot^®^ 14 SPSS. Data were expressed as mean ± SEM. Differences were tested by two-way analysis of variance (ANOVA test, factors: group x timing) completed by a Student-Newman-Keuls *post hoc* test to compare the metabosensive afferent responses to KCl and LA injections during aging process and after treadmill training (factors: age x doses). One-way ANOVA were used to compare EIF and muscle properties during aging process, while *t*-test were used to evaluate the differences after the training protocol. Results were considered statistically significant if the *p*-value fell below 0.05.

## Results

Afferents characterized as metabosensitive fibers exhibit spontaneous tonic low frequency baseline activity (4–10 Hz) under our experimental conditions. Whatever the dose and the stimulus used (LA or KCl and EIF), a significant (*p* < 0.05) increase in the raw afferent activity was recorded as compared to baseline activity within each type of muscle and experimental groups. In this experiment, in accordance with previous ones performed in the *tibialis anterior* and *soleus* muscles of Sprague-Dawley rats, we observed that the activation of muscle afferents by LA culminated for the 1 mM concentration and then declined whereas there was a relationship between the doses of KCl and the change in afferent discharge rate (Decherchi et al., 1998; Martin et al., 2009; Caron et al., 2014, 2015).

### Response to LA and KCl Injections

**FIGURE 1:**
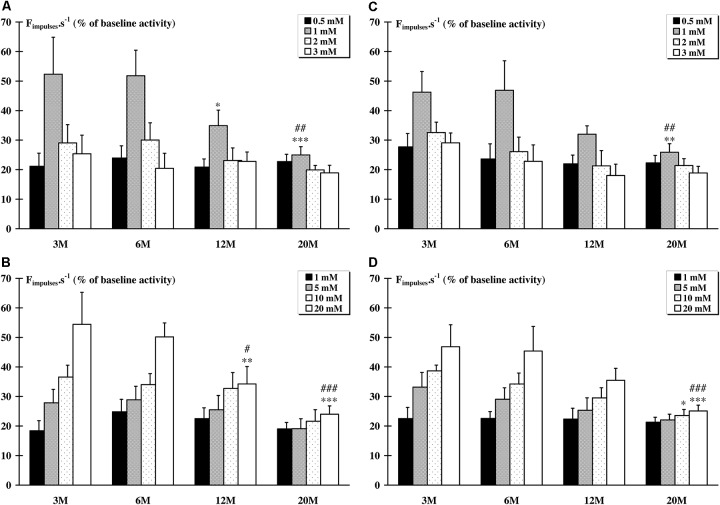
Response of metabosensitive afferents fibers to chemical injections (LA and KCl). Whatever the dose injected, a significant (*p* < 0.05) increase in the raw afferent activity was recorded as compared to baseline activity in all groups. **(A)** The response of afferent fibers from the *tibialis anterior* muscle to 1 mM LA injection was significantly (^∗^*p* < 0.05) lower in the 12 M group compared to the 3 M group, and in 20 M group compared to the 3 M (^∗∗∗^*p* < 0.001) and to the 6 M (##*p* < 0.01) groups. **(B)** The response afferent fibers from the *tibialis anterior* muscle to 20 mM KCl injection was significantly lower in the 12 M group compared to the 3 M (^∗∗^*p* < 0.01) and the 6 M (#*p* < 0.05) groups, and in the 20 M group compared to the 3 M (^∗∗∗^*p* < 0.001) and to the 6 M (###*p* < 0.001) groups. **(C)** The response of afferent fibers from the *soleus* muscle to 1 mM LA injections was significantly lower in the 20M group compared to the 3 M (^∗∗^*p* < 0.01) and the 6 M (##*p* < 0.01) groups. **(D)** The response of afferent fibers from the *soleus* muscle to 10 mM KCl injection was significantly (*p* < 0.05) lower in the 20 M group compared to the 3M group. Furthermore, the response to 20mM KCl injection was significantly lower in the 20 M group compared to the 3 M (^∗∗∗^*p* < 0.001) and to the 6 M (###*p* < 0.001), and the response to 10 mM KCl injection is significantly lower (^∗^*p* = 0.015) for the 20 M compared to 3 M group.

The pattern of responses of metabosensitive muscle afferents activated by increased interstitial concentrations of LA or KCl consisted of a burst of activity beginning within 5–10 s after the bolus injection. Recovery of baseline spontaneous discharge rate value always occurred within 3 min. For the *tibialis anterior* muscle, the responses to LA injections were decreased only for the concentration of 1 mM in animals aged of 12 (*p* < 0.05) and 20 (*p* < 0.001) months, compared to animals aged of 3 months (Figure 1A). The response to KCl injections was also decreased for the highest concentration of 20 Mm in the 12 M (*p* < 0.01) and 20 M (*p* < 0.001) groups (Figure 1B). For the *soleus* muscle, only the response to 1 mM LA concentration was decreased (*p* < 0.01) in animals from the 20 M group (Figure 1C). The response to KCl injections was also decreased for the 10 mM (*p* < 0.05) and 20 mM (*p* < 0.001) concentration in the 20 M group (Figure 1D).

**FIGURE 2:**
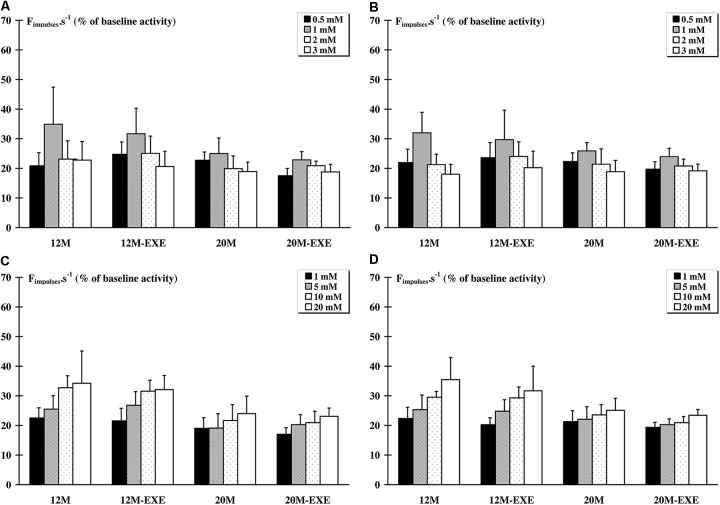
Comparison of the response of metabosensitive afferents fibers to chemical injections (LA and KCl) between non-active and active aged rats. Whatever the dose injected, a significant (*p* < 0.05) increase in the raw afferent activity was recorded as compared to baseline activity in all groups. The response of afferent fibers from *tibialis anterior* muscle to LA **(A)** and KCl **(C)** injections did not differ between old non-exercised and old trained animals. The response of *soleus* muscle afferents to LA **(B)** and to KCl **(D)** injections also did not show any difference between old non-exercised and old exercised animals.

Exercise training did not change the responses to KCl and LA injections for both muscles compared to the corresponding non-exercised animals (Figure 2).

### Response to EIF

**FIGURE 3:**
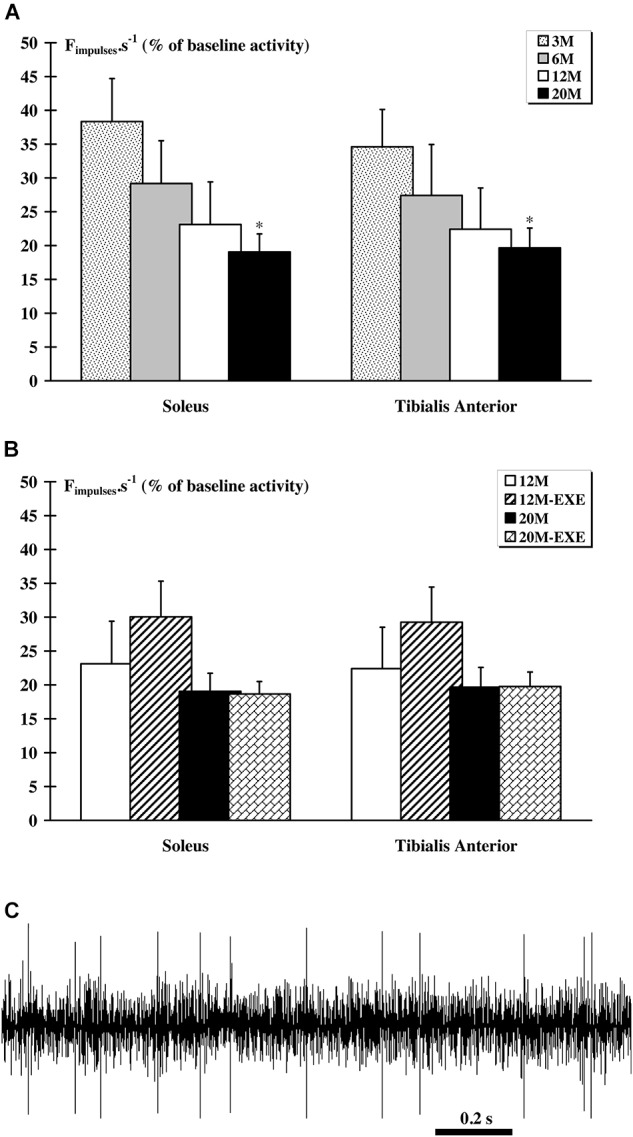
Comparison of the response of metabosensitive afferents fibers to muscle electrically-induced fatigue (EIF) between non-active and active aged rats. In all groups, a significant (*p* < 0.05) increase in activity was recorded after 3-min EIF. **(A)** In sedentary groups, the magnitude of the response to EIF decrease with age and was significantly (*p* < 0.05) lower in the 20 M group compared to the 3 M group whatever the muscle considered. **(B)** In the older animals, whatever the muscle considered and despite an increasing tendency for 12M-EXE group, no difference was noted. **(C)**. Example of extracellular recording of afferent fibers from *tibialis anterior* muscle in 12 M-EXE group.

An alteration of the response to EIF was observed with aging. Indeed, a significant (*p* < 0.05) lower afferent discharge following a 3-min EIF was observed in the 20 M groups for both muscles (Figure 3A). However, physical activity did not induce notable changes in the afferents response to EIF in aged animals although, for the both *tibialis anterior* and *soleus* muscles, there is a tendency to increase for 12M-EXE groups (Figure 3B). An example of raw recording obtained in the 12M-EXE group is showed in Figure 3C.

## Discussion

Our study reported that the response of metabosensitive afferent fibers from *tibialis anterior* and *soleus* muscles decreased with age for the doses of KCl and LA inducing the highest responses. Namely, from age of 12 months for the *tibialis anterior* muscle and 20 months for the *soleus* muscle, the responses to the dose of KCl (20 mM) and LA (1 mM) that induced the highest afferents response were reduced. Furthermore, the response to EIF was reduced for the *soleus* muscle at age of 20 months. Our results also indicated that 8 weeks of incremental treadmill running exercise did not reverse these alterations observed at 12 or 20 months.

### Effect of Aging on Metabosensitive Response.

Considering that the afferent discharge depends of the amount of metabolites released during EIF, of the number of receptors (TRPV1, ASIC3 and P2X) of terminals endings binding the metabolites endogenously released or exogenously injected, and of the number of afferents fibers on the nerve (Gao et al., 2006, 2007; Light et al., 2008), any change in any of these elements may affect the recorded response. However, it is difficult to determine the cause of this diminished response although some hypotheses can be advanced.

Anatomical studies have indicated that aging may be associated to a loss of thin myelinated and unmyelinated afferents fibers (Ceballos et al., 1999). This loss could start in the tibial nerve at 12 months of age (-59% of thin myelinated and -15% of unmyelinated fibers) and could reach 50% of fiber loss in very old animals (33 months) (Ceballos et al., 1999). However, other study founded no significant difference in the total number of neurons in lumbar dorsal root ganglia (DRG) between young and old animals (30 months) (Bergman and Ulfhake, 1998). Finally, electrophysiological study in human indicated that aging is also associated with a relative atypical unmyelinated C-fiber increase (Namer, 2010). In view to these studies, it is difficult to assume that the reduction of the number of thin myelinated and unmyelinated metabosensitive afferent fibers during aging could be responsible for the deterioration of their response.

Another explanation could be found in the receptors on the surface of the metabosensitive terminations. Wang et al. (2006) reported a decline in TRPV1 (transient receptor potential vanilloid type 1) expression in DRG and in nerve afferents innervating hind-limbs with aging but no change in TRPV1 mRNA level indicating a lower protein expression. Reduced levels of TRPV1 were also found in tibial nerve of aged animals, suggesting that TRPV1 transport is less efficient when getting older. TRPV1 level is suggested to be modulated by trophic factors, especially artemin, which receptors (GFRα3 or GDNF family receptors alpha-3) are highly co-localized with TRPV1 (Orozco et al., 2001; Elitt et al., 2006). In aged animals, because it was reported a concomitant decreased level of TRPV1 and GFRα3, and a decreased trophic support in the DRG (Wang et al., 2006), we can assume that the decrease of growth factors with aging may reduce the number of TRPV1 in metabosensitive nerve endings and consequently their responses when stimulated by metabolic agents released by muscles. However, a study indicated that the numbers of discharges induced by low pH, ATP, bradykinin, cold and heat stimuli were not different with aging (Taguchi and Mizumura, 2011).

It was reported that the conduction velocity of myelinated fibers decreases about 10–15% with age in nerve innervating the *gastrocnemius* and *soleus* muscles and in vagus nerve but not in unmyelinated fibers (Sato et al., 1985). Because metabosensitive afferents are composed of thinly myelinated and unmyelinated fibers, any change in the myelin sheath will affect the response of these afferents in a lesser extent.

Finally, because the EIF response results in metabosensitive afferent activation following muscle metabolite production, any decrease in metabolite production should affect their response. In our study, the EIF response was significantly reduced in metabosensitive afferents from *soleus* and *gastrocnemius* muscles at age of 20 months. This result suggests that the production of metabolites (lactate, K^+^, inflammatory mediators) by fatigued muscle could be affected with age. The literature described muscle anatomical changes during aging process. Indeed, aging is characterized by a progressive loss in muscle mass and a decrease number of type I (slow) and II (fast) muscle cells (muscle cell apoptosis), associated with type II cell atrophy (Lexell et al., 1988; Lexell, 1995; Narici et al., 2003). Because the metabosensitive response depends, among others, of the muscle mass and muscle phenotype (Caron et al., 2015), any change in muscle architecture may alter the release of metabolites during fatigue and consequently the afferent response to EIF.

### Effect of Exercise

Many studies show the benefit of physical exercise on pathologies or during aging. For example, it was shown the beneficial effect of aerobic exercise training on neuropathic pain (Dobson et al., 2014). It was also shown that exercise can reduce hyperglycemia and the risk to develop diabetes associated illness (Balducci et al., 2010; Li and Hondzinski, 2012), or can help to recover from peripheral nerve injury (Marqueste et al., 2004; Keeler et al., 2012; Wilhelm et al., 2012). As previously mentioned exercise training can also be an effective strategy to normalize the EPR in case of hypertension, heart failure or muscular dystrophy (Murphy et al., 2011; Smith et al., 2014). Finally, the literature reports that exercise training partially prevented the decreased of TRPV1 in DRG afferents in rats with chronic heart failure (Wang et al., 2012). Moreover, physical activity has been shown to maintain normal levels of artemin and GDNF after spinal cord injury (Detloff et al., 2014).

Assuming that alteration of the metabosensitive response with aging (20 M group) is due to a decrease in TRPV1 level and that our daily incremental treadmill exercise protocol during the last 8 weeks preceding the recordings may maintain normal level of TRPV1, we should have observed a restored response in the 12M-EXE and 20M-EXE groups. This has not been the case; the responses to KCl and LA injections, and to EIF were similar in exercised animals (12M-EXE and 20M-EXE groups) compared to non-exercised animals (12 M and 20 M groups). Only in group 12M-EXE, we observed a response that tended to increase in response to EIF. However, because of the large variability among animals, this increase was not significant. The lack of significant results could be due to the duration of the exercise we chose. Indeed, it was previously shown in rodents that a 6-month duration of aerobic exercise induced a better neuroprotection in mice model of Alzheimer disease (Garcia-Mesa et al., 2011). The authors showed that this long-lasting exercise induced benefits on synapse, redox homeostasis and general brain function. Even if our exercise protocol during the last 8 weeks preceding the recordings was not detrimental in older rats, one could hypothesize that a longer duration of exercise over months could have led to maintain an efficient afferent activity from trained muscles.

## Conclusion

Our study showed that the metabosensitive responses to metabolite injections and EIF were altered with aging and that a daily incremental treadmill exercise protocol during the last 8 weeks preceding the recordings does not restore these responses. Because these fibers are involved in regulation of sensorimotor loop, muscle pain sensation and EPR, and in physiological adjustments (Mitchell et al., 1977; Mazzone and Geraghty, 1999; Coull et al., 2003; Decherchi and Dousset, 2003; Edwards et al., 2003; Decherchi et al., 2004, 2007; Cole et al., 2010), their alteration may be responsible of some troubles observed with aging during walking and running (Markel et al., 2003; Houssiere et al., 2006), and at rest (Ng et al., 1994; Markel et al., 2003). If a repeated exercise performed when adult does not seem to reverse the effects of aging on metabosensitive afferents, it has been proved that exercise induces many positive outputs on neuromuscular functions (Koceja et al., 2004). In the future, it would be interesting to compare the type of exercise we have chosen in this study to others types of exercise or to animals that have been performed repeated exercises since they were young (i.e., throughout life).

## Author Contributions

GC, PD, and TM designed the study, carried out the analysis, analyzed the data, wrote the manuscript, and gave the final approval for publication.

## Conflict of Interest Statement

The authors declare that the research was conducted in the absence of any commercial or financial relationships that could be construed as a potential conflict of interest.

## References

[B1] Balducci S. Zanuso S. Nicolucci A. Fernando F. Cavallo S. Cardelli P. (2010). Anti-inflammatory effect of exercise training in subjects with type 2 diabetes and the metabolic syndrome is dependent on exercise modalities and independent of weight toss. *Nutr. Metab. Cardiovasc. Dis.* 20 608–617. 10.1016/j.numecd.2009.04.015 19695853

[B2] Bergman E. Ulfhake B. (1998). Loss of primary sensory neurons in the very old rat: neuron number estimates using the disector method and confocal optical sectioning. *J. Comp. Neurol.* 396 211–222. 10.1002/(SICI)1096-9861(19980629)396:2<211::AID-CNE6>3.0.CO;2-3 9634143

[B3] Caron G. Decherchi P. Marqueste T. (2015). Does metabosensitive afferent fibers activity differ from slow- and fast-twitch muscles? *Exp. Brain Res.* 233 2549–2554. 10.1007/s00221-015-4326-5 25995133

[B4] Caron G. Marqueste T. Decherchi P. (2016). Restoration of post-activation depression of the H-reflex by treadmill exercise in aged rats. *Neurobiol. Aging* 42 61–68. 10.1016/j.neurobiolaging.2016.02.022 27143422

[B5] Caron G. Rouzi T. Grelot L. Magalon G. Marqueste T. Decherchi P. (2014). Mechano- and metabosensitive alterations after injection of botulinum toxin into gastrocnemius muscle. *J. Neurosci. Res.* 92 904–914. 10.1002/jnr.23370 24615939

[B6] Ceballos D. Cuadras J. Verdu E. Navarro X. (1999). Morphometric and ultrastructural changes with ageing in mouse peripheral nerve. *J. Anat.* 195 563–576. 10.1046/j.1469-7580.1999.19540563.x 10634695 PMC1468027

[B7] Cole L. J. Farrell M. J. Gibson S. J. Egan G. F. (2010). Age-related differences in pain sensitivity and regional brain activity evoked by noxious pressure. *Neurobiol. Aging* 31 494–503. 10.1016/j.neurobiolaging.2008.04.012 18513833

[B8] Coull J. A. M. Boudreau D. Bachand K. Prescott S. A. Nault F. Sik A. (2003). Trans-synaptic shift in anion gradient in spinal lamina I neurons as a mechanism of neuropathic pain. *Nature* 424 938–942. 10.1038/nature01868 12931188

[B9] Decherchi P. Darques J. L. Jammes Y. (1998). Modifications of afferent activities from tibialis anterior muscle in rat by tendon vibrations, increase of interstitial potassium or lactate concentration and electrically-induced fatigue. *J. Peripher. Nerv. Syst.* 3 267–276. 10970127

[B10] Decherchi P. Dousset E. (2003). [Role of metabosensitive afferent fibers in neuromuscular adaptive mechanisms]. *Can. J. Neurol. Sci.* 30 91–97. 10.1017/S0317167100053348 12778889

[B11] Decherchi P. Dousset E. Grelot L. (2004). [Metabolic stability and physiological adaptation of muscle under conditions of exercise]. *Rev. Neurol.* 160 297–305. 10.1016/S0035-3787(04)70904-2 15037842

[B12] Decherchi P. Dousset E. Jammes Y. (2007). Respiratory and cardiovascular responses evoked by tibialis anterior muscle afferent fibers in rats. *Exp. Brain Res.* 183 299–312. 10.1007/s00221-007-1044-7 17643237

[B13] Decherchi P. Vuillon-Cacciutolo G. Darques J. L. Jammes Y. (2001). Changes in afferent activities from tibialis anterior muscle after nerve repair by self-anastomosis. *Muscle Nerve* 24 59–68. 10.1002/1097-4598(200101)24:1<59::AID-MUS7>3.0.CO;2-S 11150967

[B14] Detloff M. R. Smith E. J. Molina D. Q. Ganzer P. D. Houle J. D. (2014). Acute exercise prevents the development of neuropathic pain and the sprouting of non-peptidergic (GDNF- and artemin-responsive) c-fibers after spinal cord injury. *Exp. Neurol.* 255 38–48. 10.1016/j.expneurol.2014.02.013 24560714 PMC4036591

[B15] Dobson J. L. McMillan J. Li L. (2014). Benefits of exercise intervention in reducing neuropathic pain. *Front. Cell. Neurosci.* 8:102 10.3389/fncel.2014.00102 PMC3983517 24772065

[B16] Dousset E. Marqueste T. Decherchi P. Jammes Y. Grelot L. (2004). Effects of neonatal capsaicin deafferentation on neuromuscular adjustments, performance, and afferent activities from adult tibialis anterior muscle during exercise. *J. Neurosci. Res.* 76 734–741. 10.1002/jnr.20110 15139032

[B17] Edwards R. R. Fillingim R. B. Ness T. J. (2003). Age-related differences in endogenous pain modulation: a comparison of diffuse noxious inhibitory controls in healthy older and younger adults. *Pain* 101 155–165. 10.1016/S0304-3959(02)00324-X 12507710

[B18] Elitt C. M. McIlwrath S. L. Lawson J. J. Malin S. A. Molliver D. C. Cornuet P. K. (2006). Artemin overexpression in skin enhances expression of TRPV1 and TRPA1 in cutaneous sensory neurons and leads to behavioral sensitivity to heat and cold. *J. Neurosci.* 26 8578–8587. 10.1523/Jneurosci.2185-06.2006 16914684 PMC6674358

[B19] Gao Z. H. Henig O. Kehoe V. Sinoway L. I. Li J. H. (2006). Vanilloid type 1 receptor and the acid-sensing ion channel mediate acid phosphate activation of muscle afferent nerves in rats. *J. Appl. Physiol.* 100 421–426. 10.1152/japplphysiol.00659.2005 16210435

[B20] Gao Z. H. Li J. L. D. Sinoway L. I. Li J. H. (2007). Effect of muscle interstitial pH on P2X and TRPV1 receptor-mediated pressor response. *J. Appl. Physiol.* 102 2288–2293. 10.1152/japplphysiol.00161.2007 17379752

[B21] Garcia-Mesa Y. Lopez-Ramos J. C. Gimenez-Llort L. Revilla S. Guerra R. Gruart A. (2011). Physical exercise protects against Alzheimer’s disease in 3xTg-AD mice. *J. Alzheimers Dis.* 24 421–454. 10.3233/Jad-2011-101635 21297257

[B22] Greaney J. L. Schwartz C. E. Edwards D. G. Fadel P. J. Farquhar W. B. (2013). The neural interaction between the arterial baroreflex and muscle metaboreflex is preserved in older men. *Exp. Physiol.* 98 1422–1431. 10.1113/expphysiol.2013.073189 23733520

[B23] Houssiere A. Najem B. Pathak A. Xhaet O. Naeije R. van de Borne P. (2006). Chemoreflex and metaboreflex responses to static hypoxic exercise in aging humans. *Med. Sci. Sports Exerc.* 38 305–312. 10.1249/01.mss.0000187426.93464.81 16531899

[B24] Iwamoto G. A. Botterman B. R. (1985). Peripheral factors influencing expression of pressor reflex evoked by muscular-contraction. *J. Appl. Physiol.* 58 1676–1682. 10.1152/jappl.1985.58.5.1676 3997730

[B25] Kamiya A. Michikami D. Shiozawa T. Iwase S. Hayano J. Kawada T. (2004). Bed rest attenuates sympathetic and pressor responses to isometric exercise in antigravity leg muscles in humans. *Am. J. Physiol. Regul. Integr. Comp. Physiol.* 286 R844–R850. 10.1152/ajpregu.00497.2003 14701716

[B26] Keeler B. E. Liu G. Siegfried R. N. Zhukareva V. Murray M. Houle J. D. (2012). Acute and prolonged hindlimb exercise elicits different gene expression in motoneurons than sensory neurons after spinal cord injury. *Brain Res.* 1438 8–21. 10.1016/j.brainres.2011.12.015 22244304 PMC3273584

[B27] Koceja D. M. Davison E. Robertson C. T. (2004). Neuromuscular characteristics of endurance- and power-trained athletes. *Res. Q. Exerc. Sport* 75 23–30. 10.1080/02701367.2004.10609130 15532358

[B28] Laurin J. Dousset E. Decherchi P. (2010). Modulation of the spinal excitability by muscle metabosensitive afferent fibers. *J. Neurosci. Res.* 88 2755–2764. 10.1002/jnr.22432 20544822

[B29] Laurin J. Pertici V. Dousset E. Marqueste T. Decherchi P. (2015). Group Iii and Iv muscle afferents: role on central motor drive and clinical implications. *Neuroscience* 290 543–551. 10.1016/j.neuroscience.2015.01.065 25659344

[B30] Lexell J. (1995). Human aging, muscle mass, and fiber-type composition. *J. Gerontol. Ser. Biol. Sci. Med. Sci.* 50 11–16. 10.1093/gerona/50a.special_issue.11 7493202

[B31] Lexell J. Taylor C. C. Sjostrom M. (1988). What is the cause of the ageing atrophy? Total number, size and proportion of different fiber types studied in whole vastus lateralis muscle from 15- to 83-year-old men. *J. Neurol. Sci.* 84 275–294. 10.1016/0022-510X(88)90132-3 3379447

[B32] Li L. Hondzinski J. M. (2012). Select exercise modalities may reverse movement dysfunction because of peripheral neuropathy. *Exerc. Sport Sci. Rev.* 40 133–137. 10.1097/JES.0b013e31825f7483 22653276

[B33] Light A. R. Hughen R. W. Zhang J. Rainier J. Liu Z. Q. Lee J. (2008). Dorsal root ganglion neurons innervating skeletal muscle respond to physiological combinations of protons, ATP, and lactate mediated by ASIC, P2X, and TRPV1. *J. Neurophysiol.* 100 1184–1201. 10.1152/jn.01344.2007 18509077 PMC6195653

[B34] Markel T. A. Daley J. C. Hogeman C. S. Herr M. D. Khan M. H. Gray K. S. (2003). Aging and the exercise pressor reflex in humans. *Circulation* 107 675–678. 10.1161/01.Cir.0000055190.81716.Ab 12578866

[B35] Marqueste T. Alliez J. Alluin O. Jammes Y. Decherchi P. (2004). Neuromuscular rehabilitation by treadmill running or electrical stimulation after peripheral nerve injury and repair. *J. Appl. Physiol.* 96 1988–1995. 10.1152/japplphysiol.00775.2003 14634028

[B36] Martin V. Dousset E. Laurin J. Gondin J. Gautier M. Decherchi P. (2009). Group Iii and Iv muscle afferent discharge patterns after repeated lengthening and shortening actions. *Muscle Nerve* 40 827–837. 10.1002/mus.21368 19626674

[B37] Mazzone S. B. Geraghty D. P. (1999). Altered respiratory response to substance P and reduced NK1 receptor binding in the nucleus of the solitary tract of aged rats. *Brain Res.* 826 139–142. 10.1016/S0006-8993(99)01247-0 10216206

[B38] McCloskey D. I. Mitchell J. H. (1972). Reflex cardiovascular and respiratory responses originating in exercising muscle. *J. Physiol.* 224 173–186. 10.1113/jphysiol.1972.sp009887 5039977 PMC1331532

[B39] Mense S. (2009). Algesic agents exciting muscle nociceptors. *Exp. Brain Res.* 196 89–100. 10.1007/s00221-008-1674-4 19139871

[B40] Mitchell J. H. Reardon W. C. McCloskey D. I. (1977). Reflex effects on circulation and respiration from contracting skeletal muscle. *Am. J. Physiol.* 233 H374–H378. 10.1152/ajpheart.1977.233.3.H374 910926

[B41] Murphy M. N. Mizuno M. Mitchell J. H. Smith S. A. (2011). Cardiovascular regulation by skeletal muscle reflexes in health and disease. *Am. J. Physiol. Heart Circ. Physiol.* 301 H1191–H1204. 10.1152/ajpheart.00208.2011 21841019 PMC3197431

[B42] Namer B. (2010). Age related changes in human C-fiber function. *Neurosci. Lett.* 470 185–187. 10.1016/j.neulet.2009.07.023 19607877

[B43] Narici M. V. Maganaris C. N. Reeves N. D. Capodaglio P. (2003). Effect of aging on human muscle architecture. *J. Appl. Physiol.* 95 2229–2234. 10.1152/japplphysiol.00433.2003 12844499

[B44] Ng A. V. Callister R. Johnson D. G. Seals D. R. (1994). Sympathetic neural reactivity to stress does not increase with age in healthy humans. *Am. J. Physiol.* 267 H344–H353. 10.1152/ajpheart.1994.267.1.H344 8048600

[B45] Orozco O. E. Walus L. Sah D. W. Y. Sanicola M. (2001). GFRalpha3 is expressed predominantly in nociceptive sensory neurons. *Eur. J. Neurosci.* 13 2177–2182. 10.1046/j.0953-816x.2001.01596.x 11422460

[B46] Pasini E. Le Douairon Lahaye S. Flati V. Assanelli D. Corsetti G. Speca S. (2012). Effects of treadmill exercise and training frequency on anabolic signaling pathways in the skeletal muscle of aged rats. *Exp. Gerontol.* 47 23–28. 10.1016/j.exger.2011.10.003 22015326

[B47] Rotto D. M. Kaufman M. P. (1988). Effect of metabolic products of muscular-contraction on discharge of group-Iii and group-Iv afferents. *J. Appl. Physiol.* 64 2306–2313. 10.1152/jappl.1988.64.6.2306 3136123

[B48] Sato A. Sato Y. Suzuki H. (1985). Aging effects on conduction velocities of myelinated and unmyelinated fibers of peripheral-nerves. *Neurosci. Lett.* 53 15–20. 10.1016/0304-3940(85)90090-4 3991047

[B49] Sidhu S. K. Weavil J. C. Venturelli M. Rossman M. J. Gmelch B. S. Bledsoe A. D. (2015). Aging alters muscle reflex control of autonomic cardiovascular responses to rhythmic contractions in humans. *Am. J. Physiol. Heart Circ. Physiol.* 309 H1479–H1489. 10.1152/ajpheart.00433.2015 26386110 PMC4666976

[B50] Smith S. A. Downey R. M. Williamson J. W. Mizuno M. (2014). Autonomic dysfunction in muscular dystrophy: a theoretical framework for muscle reflex involvement. *Front. Physiol.* 5:47. 10.3389/fphys.2014.00047 24600397 PMC3927082

[B51] Taguchi T. Mizumura K. (2011). Augmented mechanical response of muscular thin-fiber receptors in aged rats recorded in vitro. *Eur. J. Pain* 15 351–358. 10.1016/j.ejpain.2010.08.007 20851649

[B52] Vandervoort A. A. McComas A. J. (1986). Contractile changes in opposing muscles of the human ankle joint with aging. *J. Appl. Physiol.* 61 361–367. 10.1152/jappl.1986.61.1.361 3525504

[B53] Victor R. G. Bertocci L. A. Pryor S. L. Nunnally R. L. (1988). Sympathetic-nerve discharge is coupled to muscle-cell Ph during exercise in humans. *J. Clin. Invest.* 82 1301–1305. 10.1172/Jci113730 3170747 PMC442683

[B54] Wang H. J. Li Y. L. Zucker I. H. Wang W. (2012). Exercise training prevents skeletal muscle afferent sensitization in rats with chronic heart failure. *Am. J. Physiol. Regul. Integr. Comp. Physiol.* 302 R1260–R1270. 10.1152/ajpregu.00054.2012 22496362 PMC3378347

[B55] Wang S. Y. Davis B. M. Zwick M. Waxman S. G. Albers K. M. (2006). Reduced thermal sensitivity and Nav1.8 and TRPV1 channel expression in sensory neurons of aged mice. *Neurobiol. Aging* 27 895–903. 10.1016/j.neurobiolaging.2005.04.009 15979214 PMC2841704

[B56] Wilhelm J. C. Xu M. Cucoranu D. Chmielewski S. Holmes T. Lau K. (2012). Cooperative roles of BDNF expression in neurons and schwann cells are modulated by exercise to facilitate nerve regeneration. *J. Neurosci.* 32 5002–5009. 10.1523/Jneurosci.1411-11.2012 22492055 PMC3382119

[B57] Wilson L. B. Dyke C. K. Parsons D. Wall P. T. Pawelczyk J. A. Williams R. S. (1995). Effect of skeletal muscle fiber type on the pressor response evoked by static contraction in rabbits. *J. Appl. Physiol.* 79 1744–1752. 10.1152/jappl.1995.79.5.1744 8594037

[B58] Xing J. H. Sinoway L. Li J. H. (2008). Differential responses of sensory neurones innervating glycolytic and oxidative muscle to protons and capsaicin. *J. Physiol. Lond.* 586 3245–3252. 10.1113/jphysiol.2008.154450 18450773 PMC2538781

